# Impaired Meningeal Lymphatic Drainage Aggravates LPS-Induced Neuroinflammation and Depression-Like Behaviors in Mice

**DOI:** 10.1155/mi/4288671

**Published:** 2025-08-06

**Authors:** Chonglong Shi, Yin Zhou, Qiang Zhang, Wenjie Jin, Hongquan Dong

**Affiliations:** ^1^Department of Anesthesiology, Jiangsu Province Hospital, The First Affiliated Hospital of Nanjing Medical University, Nanjing 210029, China; ^2^Department of Anesthesiology, Jiangsu Cancer Hospital and Jiangsu Institute of Cancer Research, The Affiliated Cancer Hospital of Nanjing Medical University, Nanjing 210009, China

**Keywords:** glia, meningeal lymphatic vessels, MAZ51, neuroinflammation, VEGF-C

## Abstract

**Background:** The role of meningeal lymphatic vessels (mLVs) in neurodegenerative diseases has been increasingly recognized. However, their involvement in lipopolysaccharide (LPS)-induced neuroinflammation and associated depression-like behaviors remains poorly understood. Given that impaired clearance of neurotoxic substances can prolong central nervous system (CNS) inflammation, investigating the function of mLVs in this context may offer new insights into the mechanisms underlying acute neuroinflammation and provide potential therapeutic targets.

**Methods:** First, the impact of intracerebral injection of LPS on the function of mLVs was investigated. Subsequently, the MAZ51, a VEGFR3 antagonist, was administered via intraperitoneal injection for 1 month to inhibit the development and function of mLVs, and to evaluate whether the impaired drainage function of mLVs exacerbates inflammation in the CNS caused by LPS. Finally, VEGFR3 ligand VEGF-C was administered preemptively to assess whether enhancing the function of mLVs mitigates LPS-induced inflammation and depression-like behavior.

**Results:** The mice with intracerebral injection of LPS demonstrated a substantial reduction in the transport of OVA-647 to the deep cervical lymph nodes (dCLNs) and in the coverage of Lyve-1 within the mLVs, suggesting LPS impaired the development and drainage of mLVs. Pretreatment with MAZ51 further damages the drainage function of mLVs and intensify LPS-induced activation of microglia and astrocytes. Additionally, MAZ51 led to a decrease in the protein levels of PSD95 as well in the hippocampus, which was paralleled by an elevation in TNF-α and IL-6 levels, and aggravated depression-like behavior. On the contrary, pretreatment with VEGFR3 ligand VEGF-C attenuated LPS-induced neuroinflammation, as indicated by a decrease in TNF-α, IL-6, Iba1, and GFAP expression in the hippocampus. VEGF-C treatment also significantly increased the level of PSD95 and improved depression-like behavior.

**Conclusion:** The drainage function of mLVs is pivotal in inflammation of the CNS. Enhancing meningeal lymphatic improves the development of neuroinflammation and inflammation-induced depression-like behaviors. VEGFR3 could serve as a potential therapeutic target for CNS inflammation.

## 1. Introduction

Neuroinflammation in the central nervous system (CNS) has been broadly considered as an important component of innate immunity and a major contributor to a variety of CNS disorders, including sepsis-associated encephalopathy, Alzheimer's disease, and Parkinson's disease [1, 2]. The mechanisms underlying CNS inflammation are complicated and diversified, including the activation of microglia and astrocytes, an increase in immune cells of non-CNS origin, and the accumulation of toxic macromolecules, such as damage-associated molecular patterns and pathogen-associated molecular patterns in the brain [3, 4]. An increasing amount of evidence indicates that the persistent neuroinflammation, in the form of the accumulation of these nonneuronal pro-inflammatory cells and toxic substances, becomes detrimental to neuronal cells [5, 6]. In the context of CNS injury, incomplete removal of cytotoxic factors is proposed to extend neuroinflammatory process and exacerbate the progression of CNS disease.

Traditional perspectives assert that the brain is an immune-privileged organ, partly attributable to the absence of a conventional lymphatic system; however, emerging evidence has challenged this paradigm [7, 8]. Numerous studies have demonstrated the presence of a functional lymphatic system within the meninges [9, 10]. These lymphatic vessels, distributing along the dural venous sinuses, are responsible for draining macromolecules, cellular waste, and toxic substances from the brain to peripheral organs, where they are metabolized [11]. Meningeal lymphatic vessels (mLVs) are present in the dura mater and express markers specific for lymphatic endothelial cells; vascular endothelial growth factor receptor 3 (VEGFR-3), prospero homeobox protein 1 (PROX1), and lymphatic vessel hyaluronan receptor 1 (Lyve-1) [12]. mLVs are now recognized as playing a crucial role in the drainage of cerebrospinal fluid, along with CNS waste products and antigens, from the brain to the deep cervical lymph nodes (dCLNs). Further research has confirmed that the clearance of amyloid-β, extracellular tau, and α-synuclein from the brain relies critically on the meningeal lymphatic system [13, 14]. Dysfunction of the meningeal lymphatic system may lead to inadequate clearance of neurotoxic substances and aggregate accumulate in excess, accelerating the progression of neurodegenerative diseases [15].

LPS (lipopolysaccharide), a major component of the outer membrane of Gram-negative bacteria, is widely used to model acute neuroinflammation due to its potent ability to trigger systemic inflammatory responses [16]. These responses are closely associated with neuronal injury and neurodegenerative alterations, particularly in the hippocampus—a brain region essential for emotional processing and memory consolidation [17]. Increasing evidence links neuroinflammation with the pathogenesis of depression, as patients with major depressive disorder often exhibit elevated circulating levels of inflammatory markers, such as interleukin-6 (IL-6) [18]. Mechanistically, LPS-induced glial activation and proinflammatory cytokine release contribute to oxidative stress and neuronal damage, which may underlie behavioral alterations resembling depressive or anxiety-like symptoms. While inflammation is recognized as a key factor in the development of depression, the precise mechanisms remain to be fully elucidated. Clarifying the role of mLVs in LPS-induced neuroinflammation may enhance our understanding of how the CNS regulates immune clearance during acute inflammation. This could reveal novel mechanisms by which lymphatic dysfunction contributes to prolonged neuroimmune activation and identify potential therapeutic strategies for CNS disorders.

In the present study, we investigated the effects of LPS-induced neuroinflammation on the drainage function of meningeal lymphatic and evaluated how alterations in mLVs drainage influence CNS inflammation. VEGFR3, a tyrosine kinase receptor that binds to VEGF-C, plays a critical role in promoting lymphangiogenesis by inducing the proliferation and migration of lymphatic endothelial cells [19]. To assess whether VEGFR3 represents a potential therapeutic target for CNS inflammation, we employed MAZ51, a VEGFR3 antagonist, to inhibit mLVs drainage function, and VEGF-C, an activator, to enhance it.

## 2. Materials and Methods

### 2.1. Animals

Adult male SPF-grade C57BL/6 mice (6–8 weeks old) were purchased from Beijing Vital River Laboratory Animal Technology (Beijing, China). The mice were housed in a standard laboratory setting with free access to food and water, maintained under a 12-h light/dark cycle at a constant room temperature of 22 ± 1°C. Prior to experimentation, they were acclimated to the environment for 1 week. All animal experiments were approved by the Institutional Animal Care and Use Committee of Nanjing Medical University (No. 2008037) and conducted in accordance with the National Institutes of Health Guide for the Care and Use of Laboratory Animals.

### 2.2. LPS Treatment

Mice were administered an intracerebroventricular (i.c.v.) LPS injection as previously described [20]. Briefly, mice were anesthetized via intraperitoneal injection of sodium pentobarbital (100 mg/kg) and placed in a stereotaxic apparatus (RWD). Using a 31G Hamilton syringe, 2 μg of LPS (*Escherichia coli* O111:B4, Sigma–Aldrich L2630) dissolved in 2 μL of sterile 0.9% sodium chloride solution was administered into the left ventricle over a 5 min period [20]. Control animals received an isovolumetric i.c.v. injection of 0.9% sodium chloride solution. Throughout the procedure, mice were maintained at 36 ± 1°C using a heating pad.

### 2.3. Intracisterna Magna Injection

Following anesthesia with intraperitoneal sodium pentobarbital (100 mg/kg), mice were placed in a stereotactic frame and ophthalmic solution was applied to prevent corneal drying. The neck was shaved and disinfected with 70% ethanol. A small (5 mm) skin incision was made to expose the atlanto-occipital membrane. Solutions were injected into the cerebrospinal fluid-filled cisterna magna at a rate of 2 μL/min using a 31G Hamilton syringe, which was left in place for 5 min postinjection to minimize cerebrospinal fluid leakage. After incision closure, mice received subcutaneous buprenorphine (0.5 mg/kg) and were placed on a heating pad until full recovery. Ovalbumin conjugated with Alexa Fluor 647 (OVA-647, 3 μL, 2 mg/mL, Thermo Fisher Scientific) or VEGF-Cs (3 μL, 1.33 mg/mL, Cs is Cys156Ser, a VEGFR-3-selective agonist, R&D System) was administered via this route [21]. Mice were administered 4 μg of VEGF-Cs via cisterna magna injection 1 week prior to LPS injection into the lateral ventricle [8].

### 2.4. VEGFR3 Tyrosine Kinase Inhibitor Administration

MAZ51 (Merck Millipore, Cat. No. 676492) was dissolved in dimethyl sulfoxide and administered via intraperitoneal injection at a dose of 10 mg/kg body weight for 30 consecutive days (5 days per week) [22]. Control groups received an equivalent volume of vehicle. On the 30th day, mice in both groups were further subdivided into LPS injection and 0.9% sodium chloride solution injection groups.

### 2.5. Immunofluorescence Staining

Following anesthesia, mice were transcardially perfused with phosphate-buffered saline (PBS) for 2 min, followed by 4% paraformaldehyde for 5 min. Meningeal preparation was performed as described in our previous study [21]. Briefly, the skullcap was harvested and fixed in 4% paraformaldehyde overnight at 4°C. Under a dissecting microscope, the meninges were carefully dissected from the skullcap, immersed in PBS, and transferred to a 24-well plate. The brain and dCLNs were similarly harvested, postfixed in 4% paraformaldehyde overnight at 4°C, and cryoprotected in 30% sucrose for 3 days at 4°C prior to embedding in OCT compound. For immunofluorescence, dCLNs were sectioned at 10 μm, while brain tissues were sectioned at 30 μm. Brain slices and meninges were blocked in PBS containing 0.5% Triton × -100% and 5% bovine serum albumin (BSA) for 1 h at room temperature. They were then incubated overnight at 4°C with primary antibodies diluted in PBS with 0.5% Triton × -100% and 1% BSA. Following three 15-min washes with PBS, the tissues were incubated with secondary antibodies (diluted in PBS with 0.5% Triton × -100% and 1% BSA) for 2 h at room temperature, and subsequently washed again. The brain slices and meninges were mounted on glass slides, air-dried in the dark for 10 min, and finally covered with mounting medium containing DAPI (Abcam, ab104139). Primary antibodies included rabbit anti-Lyve-1 (Abcam, ab14917, 1:200), rabbit anti-Iba1 (Wako, 019–19741, 1:500), and rabbit anti-PSD95 (Abcam, ab1238135, 1:500). Secondary antibodies included goat anti-rabbit Alexa Fluor 594 and Alexa Fluor 488 (Invitrogen, 1:500). Images were captured using a Thunder Imager fast high-resolution inverted fluorescence imaging system (LEICA, Germany). Quantitative analysis involved measuring the Lyve-1 fluorescence area in meningeal and calculating the percentage of OVA-647 fluorescence relative to the total lymph node area.

### 2.6. Enzyme-Linked Immunosorbent Assay (ELISA)

The left hippocampus of each mouse was homogenized in RIPA lysis buffer using a tissue grinder. Lysates were centrifuged, and the supernatants were collected for ELISA. Levels of TNF-α and IL-6 were measured using ELISA kits (Multiscience, China) according to the manufacturer's instructions.

### 2.7. Western Blot

The left hippocampus was homogenized in RIPA lysis buffer and centrifuged at 12,000 × *g* for 20 min at 4°C. Protein concentrations in the supernatants were measured using a bicinchoninic acid assay (Thermo Scientific). Equal amounts of protein (30 μg) were separated by SDS-PAGE and transferred onto polyvinylidene difluoride membranes (Millipore). The membranes were blocked with 5% nonfat milk for 1 h at room temperature, followed by overnight incubation at 4°C with primary antibodies, including rabbit anti-PSD95 (Abcam, ab1238135, 1:500) and rabbit anti-GAPDH (Proteintech, 1:1000). After washing, membranes were incubated with secondary antibodies (1:1000), washed again, and treated with enhanced chemiluminescence reagent. Densitometric analysis was performed using Image Lab software (Bio-Rad), and quantitative analysis was conducted using ImageJ.

### 2.8. Behavioral Tests

#### 2.8.1. Open Field Test

The open-field test was conducted in a white chamber (40 cm × 40 cm × 40 cm) illuminated by white light in a quiet, dimly lit room (30 lx). Each mouse was placed on one side of the apparatus and allowed to explore freely for 5 min. The total distance traveled, the time spent in the central area, and the distance traveled within the central area were recorded. An automatic image monitoring system (XR-XZ301, Shanghai Xinruan Information Technology Co., Ltd., Shanghai, China) was used for data tracking and analysis. To eliminate odors and prevent cross contamination between subjects, the apparatus was cleaned with 70% ethanol between trials. Behavioral scoring was conducted by an experimenter blinded to group allocation.

#### 2.8.2. Forced Swim Test (FST)

FST was carried out as previously described [23]. Mice were individually placed in a 5 L transparent glass beaker filled with 3.5 L of water maintained at 24°–25°C and illuminated with white light (210–280 Lx). Behavioral activity was recorded for a total duration of 6 min using a video tracking system. Immobility time, defined as the absence of active movements other than those required to keep the head above water, was quantified during the final 4 min of the test. Behavioral scoring was conducted by an experimenter blinded to group allocation.

#### 2.8.3. Tail Suspension Test (TST)

TST was also carried out as previously described [23].The TST was conducted using a chamber measuring 30 cm × 30 cm × 50 cm. Mice were suspended by the tail using adhesive tape affixed approximately 1 cm from the tail tip, with the tape attached to the ceiling of the chamber. The animals'behavior was recorded for 6 min using a camera positioned in front of the chamber. Immobility time, defined as the absence of initiated movements excluding those necessary for respiration and balance, was quantified during the test period. Behavioral scoring was conducted by an experimenter blinded to group allocation.

### 2.9. Statistical Analysis

Statistical analyses were performed using Prism 10.0 software (GraphPad Software, San Diego, CA). Data are presented as mean ± SD. Normality of the data was assessed using the Shapiro–Wilk test. Homogeneity of variance was evaluated with the Brown–Forsythe test. For normally distributed data with equal variances, one-way ANOVA followed by Tukey's post hoc test was used. When the assumption of equal variances was violated, Welch's ANOVA was applied with Dunnett T3 post hoc test for multiple comparisons. For two-group comparisons, unpaired two-tailed Student's *t*-test was used for normally distributed data, while the Mann–Whitney *U* test was applied for nonnormally distributed data. A *p*-value < 0.05 was considered statistically significant.

## 3. Results

### 3.1. Effects of LPS Treatment on the Drainage Function of mLVs

**Figure 1:**
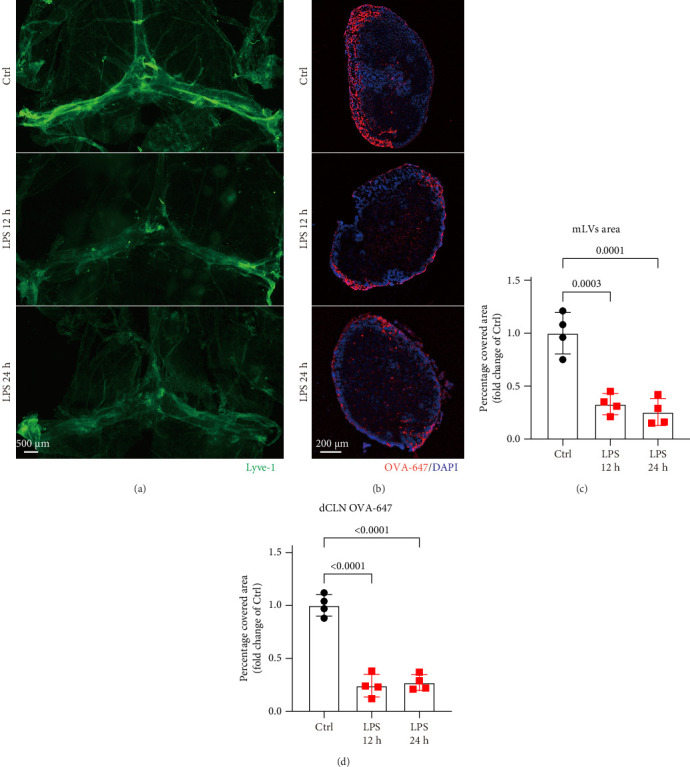
Effects of LPS treatment on the drainage function of meningeal lymphatic vessels. (A) Representative immunofluorescence images displaying the staining of Lyve-1^+^ in the meningeal whole-mounts. (B) Representative immunofluorescence images illustrating OVA-647 accumulation in dCLNs. (C) Graph depicting the percentage area of Lyve-1 coverage in the mLVs, *n* = 4. (D) Quantification of OVA-647 coverage in the mLVs, *n* = 4. The above data were analyzed using one-way ANOVA and Tukey's post hoc test. All data are expressed as the mean ± SD.

Previous studies have shown that i.c.v. injection of 2 μg of LPS induces significant neuroinflammation within 8–12 h [20]. Based on this, 2 μg of LPS was injected into the left ventricle over a 5-min period to establish a neuroinflammation model, while OVA-647 (3 μL) was injected into the cisterna magna to assess the drainage function of mLVs at 12 and 24 h post-LPS injection. Brain meninges and dCLNs were collected for analysis. As shown in Figure 1, compared to the control group, the Lyve-1-positive area—a marker of lymphatic endothelial cells—was significantly reduced in the meninges of LPS-treated mice (Figure 1A,C). Likewise, a substantial decrease in OVA-647 drainage into the dCLNs was observed following LPS treatment (Figure 1B,D). Notably, this impairment in mLVs function occurred rapidly within 12 h of LPS injection and persisted for at least 24 h. These findings suggest that LPS-induced CNS inflammation compromises the drainage capacity of mLVs.

### 3.2. VEGFR3 Inhibitor MAZ51 Impairs the Drainage Function of mLVs

**Figure 2:**
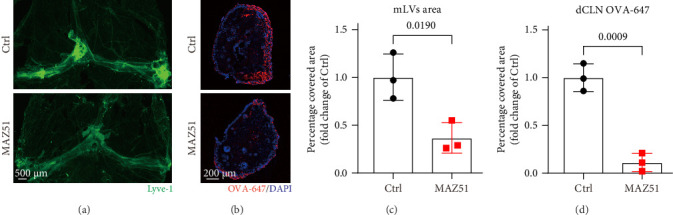
VEGFR3 inhibitor MAZ51 impairs the drainage function of mLVs. (A) Representative immunofluorescence images displaying the staining of Lyve-1^+^ in the meningeal whole-mounts. (B) Representative immunofluorescence images of OVA-647 accumulation in dCLNs. (C) Quantification of the fluorescence intensity of Lyve-1^+^ in the mLVs, *n* = 3. (D) Quantification of OVA-647 coverage in the dCLNs, *n* = 3. The above data were analyzed using unpaired two-tailed Student's *t*-test. All data are expressed as the mean ± SD.

To further investigate if the loss of mLVs integrity exacerbates neuroinflammation, we utilized MAZ51, a selective inhibitor of the VEGFR3 tyrosine kinase receptor, which has been shown to effectively suppress lymphangiogenesis [19]. Following MAZ51 treatment, mLVs exhibited significant regression, as evidenced by a marked reduction in the Lyve-1-positive area in the meninges compared to controls (Figure 2A,C). Furthermore, after OVA-647 injection into the cisterna magna, a significant decrease in OVA-647 fluorescence in the dCLNs was observed in the MAZ51-treated group (Figure 2B,D). These findings indicate that intraperitoneal administration of MAZ51 impairs lymphangiogenesis and compromises the drainage function of mLVs, suggesting a critical role of VEGFR3 signaling in maintaining mLVs integrity and function.

### 3.3. MAZ51 Treatment Exacerbates LPS-Induced Neuroinflammation

**Figure 3:**
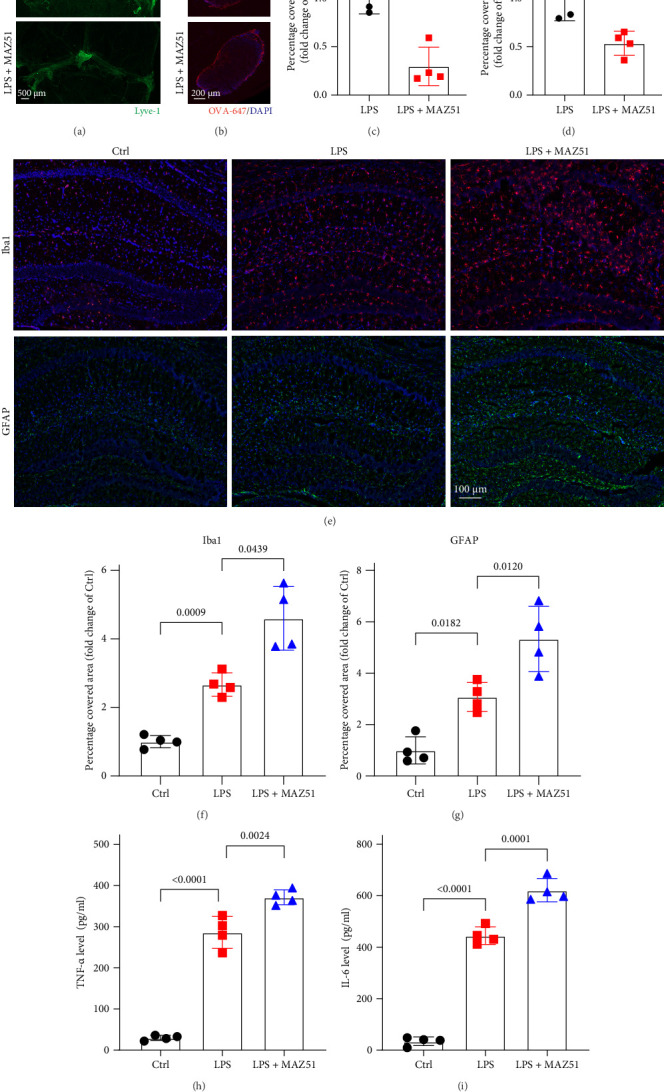
MAZ51 treatment exacerbates LPS-induced neuroinflammation. (A) Representative immunofluorescence images displaying the staining of Lyve-1^+^ in the meningeal whole-mounts. (B) Representative immunofluorescence images of OVA-647 accumulation in dCLNs. (C) Quantification of Lyve-1^+^ fluorescence intensity in mLVs, *n* = 3, the Mann–Whitney *U* test. (D) Quantification of OVA-647 fluorescence intensity in dCLNs, *n* = 3, unpaired two-tailed Student's *t*-test. (E) Immunofluorescence staining was used to detect Iba1 (red) and GFAP (green) in the hippocampus. (F) Quantitative results of the percentage of Iba1-positive area in the total area of the image, *n* = 4, Welch's ANOVA and Dunnett T3 post hoc/multiple comparison test. (G) Quantitative results of the percentage of GFAP-positive area in the total area of the image, *n* = 4, one-way ANOVA and Tukey's post hoc test. (H, I) Levels of TNF-α and IL-6 in the hippocampus, *n* = 4, one-way ANOVA and Tukey's post hoc test. All data are expressed as the mean ± SD.

As shown in Figure 3, compared with the LPS group, the significant reduction in meningeal Lyve-1 positive areas was more obvious after MAZ51 treatment (Figure 3A,C). After MAZ51 treatment, the mLVs transport function was significantly weakened, as manifested by a significant reduction in OVA-647 tracer in the dCLNs (Figure 3B,D). To investigate whether the decline in meningeal lymphatic drainage was associated with an increase in neuroinflammation, we assessed glial cell activation and measured the levels of inflammatory cytokines within the hippocampus 24 h after i.c.v. injection of LPS. Compared to the LPS group, MAZ51 treatment led to a higher percentage of Iba1 and GFAP-positive areas in the hippocampus, indicating greater activation of microglia and astrocytes (Figure 3E,F,G). As shown in Figure 3, LPS administration increased the expression levels of inflammatory cytokines, such as TNF-α and IL-6. Notably, MAZ51 treatment further exacerbated this response, as the LPS + MAZ51 group exhibited significantly higher cytokine levels than the LPS group (Figure 3H,I).

These results suggest that inhibition of VEGFR3 with MAZ51 not only impairs the drainage function of mLVs but also exacerbates neuroinflammation.

### 3.4. MAZ51 Treatment Exacerbates LPS-Induced Neuronal Damage and Depression-Like Behavior

**Figure 4:**
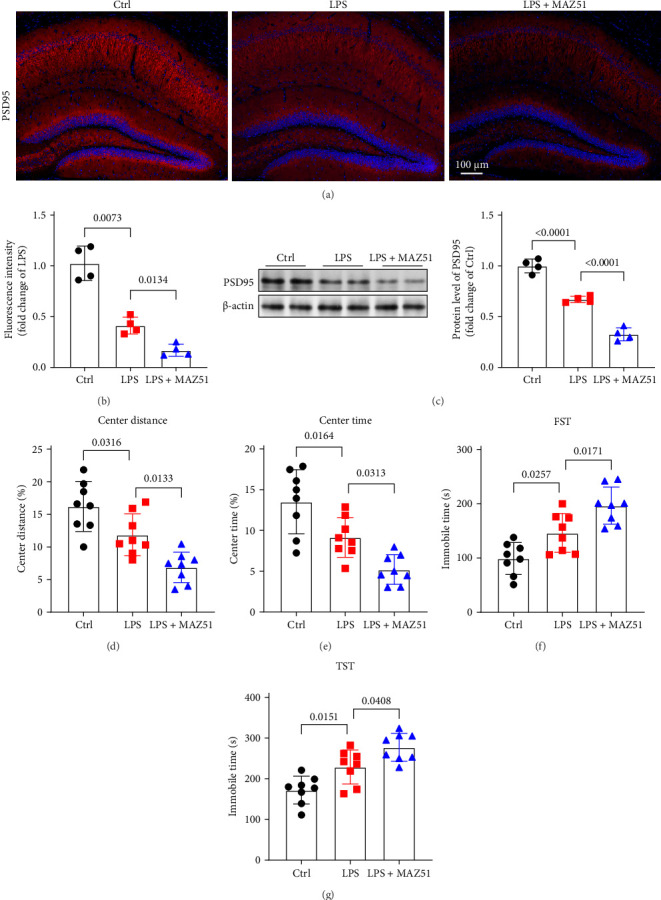
MAZ51 treatment exacerbates LPS-induced neuronal damage and depression-like behavior. (A) Representative images showing PSD95 expression in the hippocampus. (B) Quantification of the fluorescence intensity of PSD95 in the hippocampus, the value of the LPS and MAZ51 group were presented relative to the Ctrl group, with the Ctrl group designated as 1, *n* = 4. (C) The expression of PSD-95 protein in the mouse hippocampus was quantified by western blotting employing particular antibodies. And quantification of PSD95 in the hippocampus, the value of the LPS and MAZ51 group were presented relative to the Ctrl group, which was set to 1, *n* = 4, Welch's ANOVA and Dunnett T3 post hoc/multiple comparison test. (D, E) Time spent in the center and center distance in the open field test, *n* = 8. (F, G) Immobile time in the FST and TST, *n* = 8. The data from behavioral experiments were analyzed using one-way ANOVA and Tukey's post hoc test. All data are expressed as the mean ± SD.

To further investigate whether VEGFR3 inhibition exacerbates LPS-induced neuronal damage and the development of depression-like behaviors, we examined the expression of the synaptic marker PSD95 in the hippocampus and assessed behavioral changes in mice using the open-field test, FST and TST 24 h after i.c.v. injection of LPS. Similar to previous studies, after LPS treatment, the level of PSD95 in the hippocampus was significantly reduced, and behavioral performance deteriorated. As shown in Figure 4, immunofluorescent staining revealed a significant reduction in the PSD95-positive area in the MAZ51 + LPS group compared to the LPS group (Figure 4A,B). PSD95, a critical protein in the postsynaptic density, is closely associated with neuronal function, learning, and emotional regulation. Western blot analysis further confirmed the decreased expression of PSD95 in the hippocampus of the MAZ51 + LPS group, consistent with the immunofluorescence findings (Figure 4C). In accordance with previous studies showing that LPS injection induces depression-like behaviors in mice, our results demonstrated that mice in the MAZ51 + LPS group spent significantly less time and covered less distance in the central zone of the open field compared to the LPS group (Figure 4D,E). And compared with the LPS group, mice in the MAZ51 + LPS group showed longer immobility time in FST and TST (Figure 4F,G). These findings suggest that inhibition of VEGFR3 exacerbates LPS-induced neuronal dysfunction and contributes to the development of depression-like behaviors.

### 3.5. VEGF-C Alleviates LPS-Induced Central Inflammation and Brain Damage

**Figure 5:**
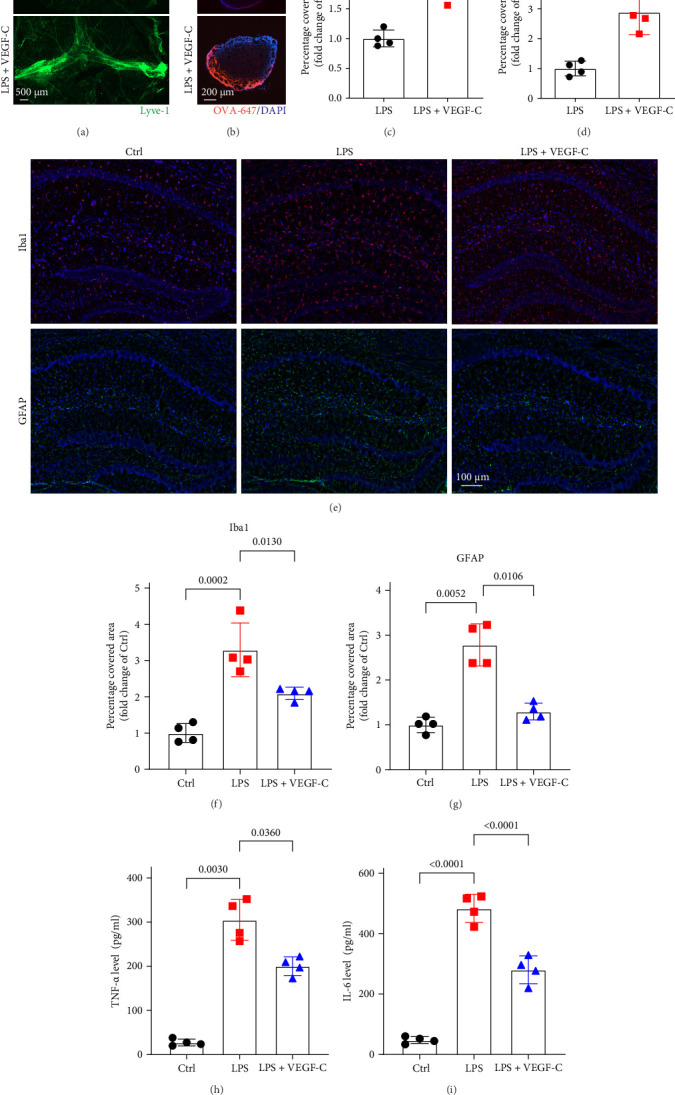
VEGF-C alleviates LPS-induced central inflammation. (A) Representative immunofluorescence micrographs showing Lyve-1^+^ staining in meningeal whole-mount preparations. (B) Representative immunofluorescence micrographs showing OVA-647 accumulation in dCLNs. (C, D) Quantification of Lyve-1^+^ fluorescence intensity in mLVs and OVA-647 fluorescence intensity in dCLNs, *n* = 3, unpaired two-tailed Student's *t*-test. (E) Representative immunofluorescence images of Iba1 (red) and GFAP (green) in the hippocampus. (F, G) Quantification of Iba1 and GFAP in the hippocampus, *n* = 4, Iba1: one-way ANOVA and Tukey's post hoc test. GFAP: Welch's ANOVA and Dunnett T3 post hoc/multiple comparison test. (H, I) ELISA was used to detect the levels of TNF-α and IL-6 in the hippocampus, *n* = 4, TNF-α: Welch's ANOVA and Dunnett T3 post hoc/multiple comparison test, IL-6: one-way ANOVA and Tukey's post hoc test. All data are expressed as the mean ± SD.

**Figure 6:**
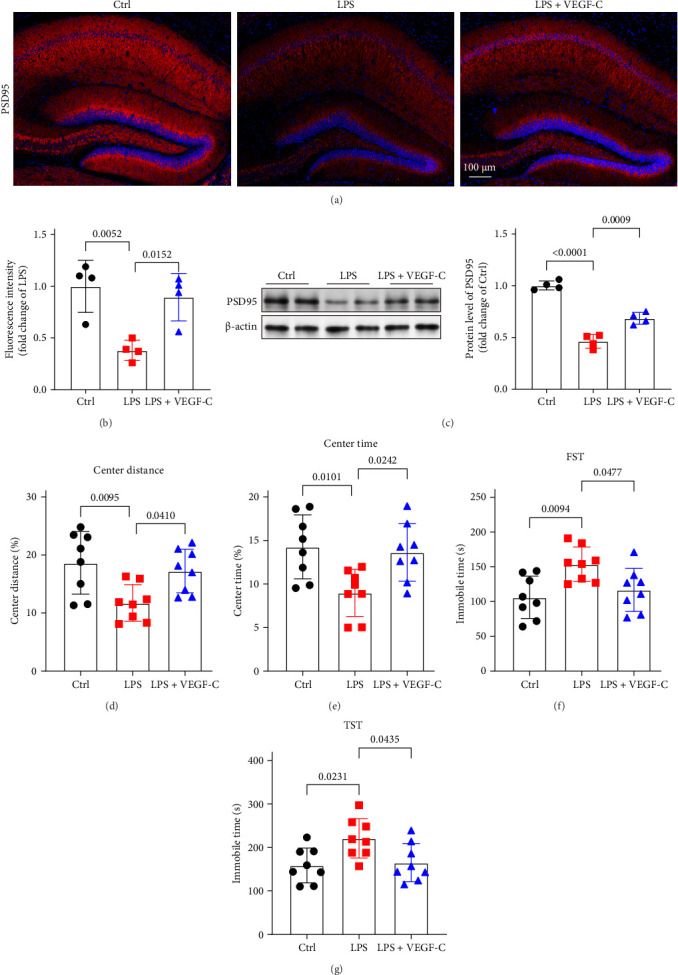
VEGF-C alleviates LPS-induced brain damage. (A) Representative immunofluorescence images of PSD95 (red) in the hippocampus. (B) Quantification of PSD95 in the hippocampus, *n* = 4. (C) Western blot was used to detect the levels of protein PSD95 in the hippocampus. Expression of PSD-95 was quantified and the value of VEGF-C and LPS group were then expressed relative to the Ctrl group, which was set to 1, *n* = 4. (D, E) Time spent in the center and center distance in the open field test, *n* = 8. (F, G) Immobile time in the FST and TST, *n* = 8. The above data were analyzed using one-way ANOVA and Tukey's post hoc test. All data are expressed as the mean ± SD.

To gain a deeper understanding of the functional impact of impaired meningeal lymphatic function in LPS-induced neuroinflammation, we explored whether enhancing the function of mLVs through VEGFR3 activation could mitigate LPS-induced central inflammation and brain damage. Mice were administered 4 μg of VEGF-Cs via cisterna magna injection 1 week prior to LPS injection into the lateral ventricle [8], and analyses were performed 24 h after LPS administration. Our results revealed that after VEGF-Cs treatment, mLVs exhibited significant enhancement, as evidenced by a marked increase in the Lyve-1-positive area in the meninges, and a significant elevation in OVA-647 fluorescence in the dCLNs compared to LPS group (Figure 5A–D). And compared to the LPS-only group, VEGF-Cs treatment significantly reduced immunofluorescence staining of Iba1 and GFAP in the hippocampus, indicating reduced microglial and astrocytic activation. Moreover, the levels of inflammatory cytokines, including TNF-α and IL-6, were markedly decreased (Figure 5E–I. These findings suggest that enhancing the drainage function of mLVs through VEGF-C injection effectively alleviates neuroinflammation induced by LPS. We further assessed synaptic function by measuring PSD95 protein expression in the hippocampus. Interestingly, VEGF-C treatment significantly increased PSD95 levels (Figure 6A–C), suggesting that VEGF-C can improve synaptic plasticity. Behavioral tests also revealed that VEGF-C treatment significantly enhanced exploration time and distance in the central area of the open field test (Figure 6D,E). And compared with the LPS group, mice in the VEGF-C + LPS group showed shorter periods of immobility time in forced swimming test and TST, which indicating that VEGF-C mitigates LPS-induced depressive-like behaviors (Figure 6F,G). These results demonstrate that VEGFR3 activation through VEGF-C administration alleviates LPS-induced neuroinflammation, improves synaptic function, and reduces depression-like behaviors.

## 4. Discussion

Neuroinflammation is increasingly recognized as a critical driver of neurodegenerative processes. Emerging evidence suggests that mLVs play a pivotal role in modulating neuroinflammation, contributing to the maintenance of CNS homeostasis [24, 25]. For instance, studies have demonstrated that mLVs facilitate the clearance of immune cells and macromolecules from the cerebrospinal fluid into the dCLNs, a process essential for mitigating brain inflammation and preserving neurological function [26]. In this study, we investigated the role of mLVs in modulating LPS-induced central inflammation. Our findings revealed that mLVs sustain immediate damage following i.c.v. LPS injection, with this impairment persisting over time. Moreover, we demonstrated that cisterna magna administration of VEGF-C enhances mLVs drainage function in LPS-treated mice, attenuates microglial and astrocyte activation, and alleviates LPS-induced depression-like behavior. Conversely, intraperitoneal administration of MAZ51 exacerbated mLVs damage, further amplifying neuroinflammation and depressive-like symptoms. These findings provide novel insights into the interplay between mLVs drainage, neuroinflammation, and brain injury, underscoring the therapeutic potential of targeting VEGFR3 signaling to mitigate neuroinflammatory conditions.

CNS diseases represent the leading cause of disability and the second most common cause of mortality worldwide [27]. Most CNS disorders are characterized by persistent neuroinflammation and an impaired ability to clear neurotoxic debris, both of which contribute to disease progression and neuronal dysfunction [28]. Neuroinflammation has become increasingly recognized as a critical target for the amelioration of neurological diseases, as it plays a central role in the progression of conditions that lead to significant clinical disability [29]. Recently, the meningeal lymphatic system has been recognized as a crucial pathway for CNS drainage, playing an essential role in the clearance of waste products and immune cells from the brain [30]. Several recent studies have emphasized the critical role of the meningeal lymphatic system in regulating inflammatory processes and facilitating the clearance of brain tumors in the CNS. In mouse models of Alzheimer's disease, the meningeal lymphatics were involved in the removal of macromolecules and metabolic waste from the brain, helping to maintain CNS health. This clearance process also contributed to significant improvements in learning and memory performance [31]. Previous studies have demonstrated that preexisting lymphatic dysfunction before traumatic brain injury leads to the upregulation of genes associated with neuroinflammation and complement pathway activation at the 24-h postinjury interval. This suggests that impaired lymphatic drainage may exacerbate the inflammatory response following traumatic brain injury, contributing to prolonged brain injury and dysfunction [32]. In the context of subchronic variable stress, intracisternal delivery of adeno-associated virus serotype 1 vector encoding vascular endothelial growth factor C, which enhances meningeal lymphatics, was shown to alleviate stress-induced depression-like behaviors in female mice. This suggests that promoting meningeal lymphatic function could offer a therapeutic approach for mitigating stress-related mood disorders [33].

However, the role of mLVs in LPS-induced central inflammation remains not fully understood. Our results demonstrated that i.c.v. injection of LPS significantly impaired mLVs function, as evidenced by the reduced Lyve-1-positive area in the meninges and diminished OVA-647 drainage to the dCLNs. These findings are consistent with prior studies, which suggest that acute inflammation can compromise the integrity and drainage capacity of mLVs, potentially exacerbating neuroinflammation [34]. The rapid onset of this impairment, observed as early as 12 h post-LPS injection, underscores the vulnerability of mLVs to inflammatory insults. Notably, this dysfunction persisted for at least 24 h, indicating that the impairment of mLVs is sustained over time during central inflammation, potentially contributing to prolonged neuroinflammation and brain injury.

To further investigate the effects of mLVs dysfunction, we employed MAZ51, a VEGFR3 tyrosine kinase inhibitor, to inhibit lymphangiogenesis and mLVs function. Prolonged VEGFR3 inhibition led to the regression of mLVs lymphangiogenesis, as evidenced by a reduction in Lyve-1-positive areas in the transverse and superior sagittal sinuses. This damage was accompanied by exacerbated LPS-induced neuroinflammation, with significant increases in microglial (Iba1) and astrocytic (GFAP) activation in the hippocampus. Moreover, hippocampal levels of inflammatory mediators, particularly TNF-α and IL-6, were markedly elevated in the MAZ51 group compared to the LPS group. In our previous studies, we also found that ligation of the dCLNs exacerbated LPS-induced central inflammation following intraperitoneal injection. These findings underscore the importance of mLVs in regulating neuroinflammation and highlight the potential consequences of their dysfunction [21]. However, cerebrospinal fluid is also drained to the extracranial lymph nodes through the perineural and perivascular subarachnoid spaces surrounding cranial nerves and cranial arteries [35]. Therefore, the method of dCLNs ligation, which simulates impaired drainage function of the meningeal lymphatic system, may not fully capture the extent of dysfunction in these pathways. To address this limitation, we employed MAZ51 in the present study to directly inhibit the development of mLVs and observe its impact on neuroinflammation. This approach allowed for a more targeted investigation into the effects of mLVs dysfunction on neuroinflammation. Behaviorally, MAZ51-treated mice exhibited worsened depression-like behaviors, as evidenced by reduced exploration time and distance in the central area of the open field test. These findings suggest that VEGFR3 inhibition not only impairs mLVs function but also exacerbates LPS-induced neuronal damage and behavioral deficits. This highlights the critical role of mLVs in maintaining brain homeostasis during inflammation and underscores the potential therapeutic value of targeting VEGFR3 signaling in neuroinflammatory conditions.

Conversely, activation of VEGFR3 through the injection of VEGF-C demonstrated protective effects against LPS-induced central inflammation and brain injury. Previous studies have shown that VEGF-C can enhance lymphatic drainage by promoting the development and remodeling of mLVs [31]. This enhancement of mLVs function facilitates the removal of neurotoxic debris and immune cells, thereby mitigating neuroinflammation and reducing neuronal damage, which highlights the therapeutic potential of VEGF-C in neuroinflammatory disorders [36]. We also confirmed that the administration of VEGF-C via cisterna magna injection increased the transport of the tracer OVA-647 to the dCLNs. This restoration of mLVs integrity was closely associated with the inhibition of microglial and astrocytic activation, reduced levels of pro-inflammatory cytokines in the hippocampus, and enhanced expression of the synaptic marker PSD95. Interestingly, in a traumatic brain injury model, exogenous VEGF-C administration was shown to promote the remodeling of mLVs, improve their drainage function, reduce inflammatory responses, and alleviate cognitive dysfunction [32]. These findings underscore the potential of VEGF-C as a therapeutic strategy for modulating neuroinflammation and improving brain function in various neurological conditions [32]. In our study, mice treated with VEGF-C also exhibited significant improvements in anxiety and depressive behaviors, suggesting that overexpression of VEGF-C could have a beneficial effect on behavioral deficits. These results highlight that VEGFR3 activation has neuroprotective effects by counteracting mLVs dysfunction, improving synaptic plasticity, and inflammatory responses. VEGF-C could effectively mitigate the pathological consequences of LPS-induced central inflammation by enhancing mLVs drainage capacity. Strategies involving VEGFR3 activation or its antagonists may offer promising approaches to improving meningeal lymphatic function, reducing neuroinflammation, and protecting neurons.

The findings of this study have broad implications for neuroinflammatory and neurodegenerative diseases. The dysfunction of mLVs has been implicated in the accumulation of toxic proteins, such as amyloid-β, tau and α-synuclein, which are hallmarks of Alzheimer's and Parkinson's diseases. By enhancing mLVs function and improving the clearance of these toxic proteins, therapies targeting VEGFR3 signaling could provide a novel approach to mitigating the progression of such diseases. This research opens up new avenues for treatment strategies that aim to restore proper lymphatic drainage in the brain and potentially slow or prevent neurodegenerative processes [24, 37, 38]. Impaired mLVs function can obstruct the drainage of neurotoxic proteins to peripheral lymph nodes, resulting in their accumulation in the brain and subsequent neuroinflammation. Furthermore, neuroinflammation can not only arise from dysfunctional meningeal lymphatics but also impact their structure and function [21]. Our results extend these findings to acute inflammatory conditions, showing that impaired mLVs function can worsen neuroinflammation and neuronal damage. The ability of VEGFR3 activation to restore mLVs function and reduce inflammation suggests that enhancing mLVs drainage may offer a promising therapeutic strategy for various CNS diseases. This study has several limitations. First, the use of intraperitoneal MAZ51 to inhibit VEGFR3 may block meningeal lymphatic function indirectly. It remains unclear whether MAZ51 exerts its effects directly on the mLVs or through systemic or alternative pathways. Second, although we focused on the drainage function of mLVs, cerebrospinal fluid outflow also occurs via other routes, such as perineural and perivascular spaces surrounding cranial nerves and arteries. To address this, we plan to employ Visudyne—a photoconvertible compound shown to effectively ablate lymphatic vessels—by delivering it into the CSF to establish a more targeted and controlled model of impaired mLVs function.

## 5. Conclusion

This study demonstrates that mLVs dysfunction exacerbates LPS-induced neuroinflammation, neuronal damage, and depression-like behaviors, while activation of VEGFR3 with VEGF-C enhanced mLVs function, reduced inflammation, and improved synaptic integrity and behavioral outcomes. These findings highlight the critical role of mLVs in maintaining CNS homeostasis, and suggest that targeting VEGFR3 signaling may offer a promising therapeutic strategy for mitigating neuroinflammatory conditions and neurodegenerative diseases.

## Data Availability

The data supporting the findings of this study can be obtained from the corresponding authors upon request.
